# The Impact of Ethnicity on Athlete ECG Interpretation: A Systematic Review

**DOI:** 10.3390/jcdd9060183

**Published:** 2022-06-08

**Authors:** Angus J. Davis, Christopher Semsarian, John W. Orchard, Andre La Gerche, Jessica J. Orchard

**Affiliations:** 1 Agnes Ginges Centre for Molecular Cardiology, Centenary Institute, Sydney 2050, Australia; christopher.semsarian@sydney.edu.au (C.S.); jessica.orchard@sydney.edu.au (J.J.O.); 2 Faculty of Medicine and Health, The University of Sydney, Sydney 2006, Australia; john.orchard@sydney.edu.au; 3 Baker Heart and Diabetes Institute, Melbourne 3004, Australia; andre.lagerche@baker.edu.au

**Keywords:** athlete, electrocardiogram interpretation, ethnicity, screening, preventative cardiology

## Abstract

Athlete ECG interpretation criteria have been developed and refined from research in athlete populations; however, current guidelines are based on available data primarily from Caucasian and Black athletes. This study aimed to assess the impact of ethnicity on ECG interpretation in athletes. A systematic review was conducted of the MEDLINE, EMBASE, Scopus, SPORTDiscus, and Web of Science databases, for papers that assessed athlete screening ECGs and compared findings on the basis of ethnicity. Fifty-one papers which compared ECGs from various ethnicities were included. Most studies assessed Black athletes against Caucasian athletes and found a greater prevalence of T-wave inversion (TWI) (2.6–22.8% vs. 0–5.0%) and anterior TWI (3.7–14.3% vs. 0.6–2.0%). Black athlete subgroups in Africa had TWI (20–40%) and anterior TWI (4.3–18.7%) at a higher prevalence than other Black athletes. Athletes who were defined as mixed-race, Asian, and Pacific Islander are potentially more like Black athletes than Caucasian athletes. Black ethnicity is known to have an impact on the accurate interpretation of athlete ECGs; however, there is nuance related to origin of both parents. Asian and Pacific Islander origin also may impact athlete ECG interpretation. Further research is required to assist in distinguishing abnormal and normal athlete ECGs in different ethnic populations.

## 1. Introduction

Athletes are known to experience training-related adaptations to exercise, which can vary according to ethnicity [1]. Training-related physiological changes are more common in athletes exercising intensively at least 4–8 h per week [1]. Despite the high level of physical fitness of athletes, sudden cardiac death is a rare but tragic occurrence that is 2.5 times more likely in elite athlete populations than age-matched young populations [2]. Preparticipation cardiac screening of athletes is, therefore, now commonly recommended at the elite level, with the aim of minimizing the risk of sudden cardiac death [3,4]. Screening generally includes a history and physical examination and a resting 12-lead electrocardiogram (ECG) [3,4].

Athlete ECG interpretation has improved significantly over the last two decades. Athletes have greater cardiac adaption than nonathletes due to intense physical activity [1]. This cardiac adaption is reflected in ECGs and their interpretation [1]. However, the interpretation of ECGs has changed over time [5]. The 2005 European Society of Cardiology (ESC) recommendations noted ECG phenotypes warranting further investigation. These recommendations were primarily created by a small cohort of cardiologists according to their experience [6]. The 2010 ESC recommendations characterized ECG changes into “training-related” and “training-unrelated” [7]. This was in response to the previous recommendations which classed 50% of athletes as abnormal, resulting in too high a false positive rate [5]. Despite the improvement in detecting disease, the ESC criteria were ineffective in the assessment of Black athletes [5]. The Seattle criteria (2013) categorized Black athletes with convex ST segment elevation with T-wave inversion (TWI) in leads V1–V4 as a “normal finding” [8]. The Black athlete variant was classed as a “borderline variant” in the Refined Criteria (2014) [9] before being added as a “normal ECG finding” in the International Criteria for Athlete ECG interpretation (2017) [1].

While the Refined Criteria (2014) and the International Criteria (2017) added borderline ECG findings, as well as reduced the rate of false positives [5], the Black athlete variant is the only ethnic variant in the criteria. An expert opinion piece from 2020 suggests that there may be further variation in Black athletes, as well as adaptation in other ethnic populations [10]. Therefore, we sought to systematically review current papers to assess the impact of ethnicity on the interpretation of athlete ECGs.

## 2. Materials and Methods

The systematic review was performed following guidelines provided by the Cochrane Handbook and the Preferred Reporting Items for Systematic Reviews and Meta-Analyses (PRISMA) [11]. Meta-analysis was not possible due to the heterogeneity of study outcome measures. The protocol was prospectively registered on PROSPERO with ID number CRD42021277646.

### 2.1. Literature Search

MEDLINE, EMBASE, Scopus, SPORTDiscus, and Web of Science databases were searched from 1 January 2000 to 15 September 2021 with English language restrictions. Medical subject heading and free search terms were used individually and combined and included the terms athlete OR sportsperson AND ethnicity OR race AND ECG OR EKG OR electrocardiogram. A full list of search terms is provided in Supplementary Appendix SA. We also hand-searched reference lists from screened articles and searched for published authors to identify other relevant studies.

### 2.2. Study Selection

Studies were included where study participants were identified as athletes by the article, comparisons between athletes were made on the basis of ethnicity, athlete screening ECGs were assessed by the study, and rates of abnormal ECGs were recorded.

Studies were included that assessed ECGs of a non-Caucasian athlete group without a control race, but not if the only racial group assessed was Caucasian.

Studies were excluded if most participants were under 16 years old, if they were based on athletes with known cardiovascular disease, or if they were review articles or conference abstracts only. Abstract and full-text screening was conducted using Covidence independently by two researchers (A.J.D. and J.J.O.). Conflicts were resolved through discussion, and, if agreement could not be reached, a third researcher (J.W.O.) independently reviewed the article.

### 2.3. Data Extraction

We developed a data extraction sheet, which was pilot-tested on four randomly selected included studies and refined accordingly. One author (A.J.D.) extracted the data from the included studies, and a second author (J.J.O.) checked the extracted data. Disagreements were resolved by discussion between the two authors. The data extracted included author, year of publication, study design, characteristics of participants (ethnicity, number of participants, mean age, and gender), ECG features noted in the study, and key conclusions from the study.

### 2.4. Quality and Risk of Bias Assessment

To assess the quality of selected studies, the reviewers used the National Heart, Lung, and Blood Institute quality assessment tool for observational cohort and cross-sectional studies [12]. Fourteen questions were asked of each paper; results were assessed by one author (A.J.D.) and checked by a second author (J.J.O.). Papers were judged as “good”, “fair”, or “poor”.

## 3. Results

### 3.1. Search Results

**Figure 1:**
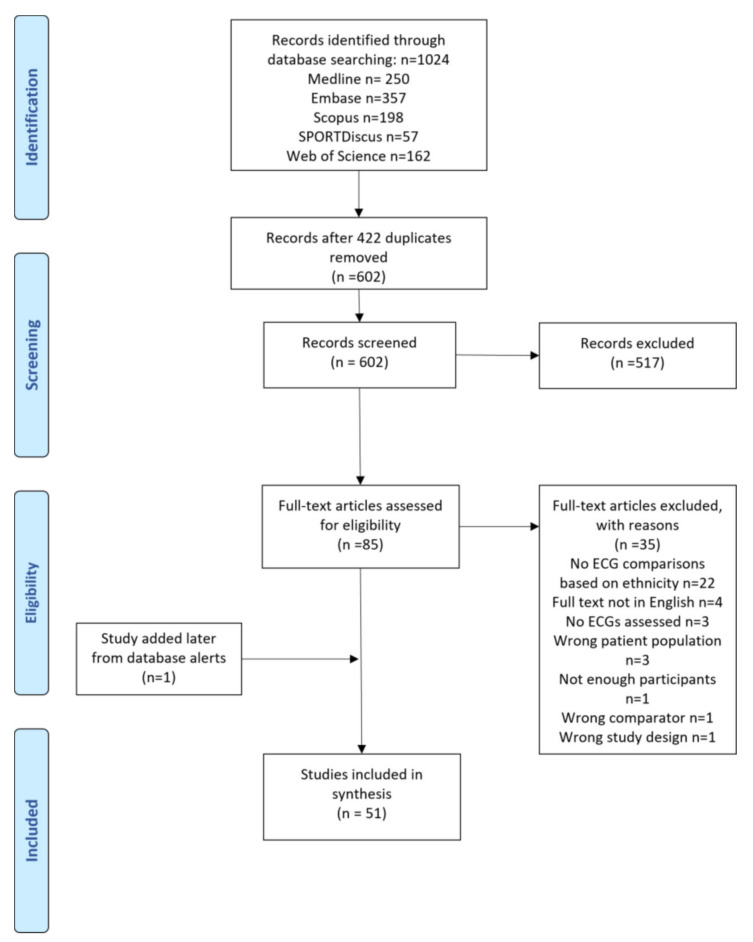
Flow diagram of included studies.

The electronic search strategy identified 602 records from the five electronic databases searched after duplicities were removed. After title and abstract review, 85 studies were included for full-text screening. Through full-text screening, 35 studies were excluded, leaving 50 studies that were included in the analysis. One further study was added after the original search as a highly relevant study that only appeared as an abstract on the extraction date but with the full paper published during our analysis. Therefore, a total of 51 papers were included in the final analysis. The search results are detailed in Figure 1.

### 3.2. Characteristics of Studies

Most studies in this paper were cross-sectional studies (48/51, 94.1%) with three papers that were cohort studies. Across all studies, there were 65,629 athlete participants whose ages ranged from 16–30 years. However, many athletes were from overlapping cohorts. Five papers included athletes screened by the Cardiac Risk in the Young organization in the UK [9,13,14,15,16], and some cohorts may have been included in more than one study from this group. Details of the study characteristics appear in Supplementary Table S1.

### 3.3. Quality Assessment

Quality assessment judged all studies as “fair”. No studies were excluded for quality. Details appear in Supplementary Appendix SB.

### 3.4. Athlete ECG Features by Ethnicity

#### 3.4.1. Groups with Significant Evidence Suggesting Variation from Caucasian Athletes

##### Black Athletes

Black athletes were the most common distinct ethnic group of athletes referred to in the literature. Black athletes were either undefined or had a definition related to African descent, including African American or Afro-Caribbean. Darker-skinned Asian, Middle Eastern, or Pacific Islander athletes were not referred to as Black in the surveyed literature. Of the 51 papers included in analysis, 32 papers discussed Black athletes. Of the included papers, 26 found that there was a significant difference between Black athlete and Caucasian athlete ECGs [9,13,14,15,16,17,18,19,20,21,22,23,24,25,26,27,28,29,30,31,32,33,34,35,36,37], but six papers concluded there were no significant differences between athletes [38,39,40,41,42,43]. Of the 26 papers that reported differences, 19 identified specific differences between groups of athletes, while the other seven papers noted an overall rate of abnormal ECGs noted by various criteria. ECG differences are summarized in Table 1.

The most common ECG difference between Black and Caucasian athletes in the papers analyzed was TWI. A higher prevalence of TWI in Black athletes vs. Caucasian athlete populations was found in 10 papers, varying from 2.6% to 22.8% of Black athletes vs. 0% to 5.0% of Caucasian athletes [15,16,17,22,24,25,27,28,32,37]. Anterior TWI beyond V2 was noted as more common in Black athletes than Caucasian athletes in six papers, found in 3.7%–14.3% of Black athletes and 0.6%–2.0% in Caucasian athletes [15,16,27,28,32,36]. Inferior TWI, in lead II, and aVF was found to be more common in five studies, ranging from 1.3% to 6.1% in Black athletes and from 0.5% to 1.7% in Caucasian athletes [15,16,27,28,33]. Lateral T-wave inversion, leads V5, V6, and I, and aVL were found as more common in five papers, ranging from 0.9% to 6.1% in Black athletes and from 0% to 0.3% in Caucasian athletes [15,16,27,28,36]. ST elevation differences were identified in eight papers, commonly found in the anterior and lateral leads, ranging from 11% to 85% in Black athletes and from 1% to 61% in Caucasian athletes [15,16,17,23,28,30,32,37].

Black athletes were different from Caucasian athletes in other ECG findings; however, there was variability in the significance of those differences. Early repolarization was noted as significantly more common in Black athletes than Caucasian athletes in four papers (34.7–72.6% vs. 17–58.3%) [16,29,31,35], but insignificant in one other [40]. Left-ventricular hypertrophy (LVH), as defined by each paper, was noted as more likely in Black athletes than Caucasian athletes in five papers (2–68% vs. 0.2–40%) [17,22,24,25,35], but not significant in two papers [19,40] and more likely in Caucasian athletes in one paper (17.2% vs. 25.6%; *p* = 0.001) [28]. T-wave inversion [39] and ST elevation [41,43] were also noted as not significantly different between ethnicities in a small number of papers.

Some papers were not specific to ECG differences and compared ethnicities on the basis of percentage abnormal according to the criteria available at the time of the study. This method was used for nine papers, of which eight found that Black athletes were more likely than Caucasian athletes to have an abnormal ECG according to the criteria used, with the percentage abnormality ranging from 11.5% to 57.7% in Black athletes and from 5.3% to 21.3% in Caucasian athletes [9,13,20,24,25,26,33,34,36]. Two papers found nonsignificant differences between ethnicities according to the criteria [38,44].

There were other less common differences found between Black athletes and Caucasian athletes that appeared in one or two papers. Right-atrial enlargement was noted in two papers [28,36] as more prominent in Black athletes (3.8–4.7%) than Caucasian athletes (0–0.7%), while left-atrial enlargement was noted in one paper [27], occurring in Black athletes (5.7%) more than Caucasian athletes (0.9%). There was some disagreement as to whether Black athletes have shorter QTc intervals [14,21] or longer QTc intervals [18].

##### West African Athletes

West African athletes were more likely than other Black athlete populations to have the “Black athlete” variations. Of the nine papers that specifically assessed West African athletes, six papers assessed West African athletes independently [45,46,47,48,49,50] while the other three papers compared the West African athletes to other athlete populations [17,33,34]. The differences are presented in Table 2. TWI was reported in four papers and, generally, TWI ranged from 6.4% to 40%, anterior TWI ranged from 4.3% to 18.7%, and inferior TWI ranged from 0.5% to 8.8% [33,46,47,49]. ST elevation was reported in three papers and ranged from 29.9% to 47% [45,46,47]. Ilodibia et al. (2021) reported Nigerian athletes to have a high prevalence of LVH (40.3%), early repolarization pattern (51.3%), and dome-shaped ST-segment elevation with TWI (27.3%) [45]. Percentage abnormal was reported by five papers, ranging from 8% to 25.8% [46,47,48,49,50].

##### African Athletes

A total of four papers identified their Black athletes as “African” [51,52,53,54]. While this was only seen in a small number of papers, African athletes appeared to have similar ECG differences to the Black athlete population. The differences are recorded in Table 3 with African athletes compared to Caucasian, Arab, or both. General TWI was found as a common ECG difference in three papers, with 7–14% of African athletes having TWI vs. 1–3% of Caucasian and Arabic athletes [51,53,54]. Two papers reported anterior TWI rates, which ranged from 2.6–23.2% of African athletes and 1–10.3% of Arabic athletes [52,53]. Lateral TWI was found in the same two papers in 3.3–3.5% of African athletes and 1%–1.4% of Arabic athletes [52,53]. The percentage of abnormal ECG findings was noted in two papers [52,54]. In one paper using the Refined Criteria (2014) [54], African athletes had a 20% rate of abnormal ECGs, while Caucasian athletes had a 6.9% rate of abnormal ECGs. In two more recent papers using the International Criteria (2017) [52,53], African athletes had a 10.5–15% rate of abnormal ECGs, while Arabic athletes had a 4.3–6.1% rate of abnormal ECGs. ST elevation and LVH were found to be more common in African athletes than Caucasian athletes in one paper at 91% vs. 56% (*p* = 0.001) for ST elevation and 89% vs. 42% (*p* = 0.001) for LVH [51].

##### East African Athletes

East African athletes were only specifically assessed in one paper and did not significantly vary from Black athletes [33].

##### Middle African Athletes

Middle African athletes, from Central or Southern Africa, were assessed in three studies, two studies by comparison to other ethnicities [33,55] and one study which assessed Middle African athletes independently [56]. These differences are detailed in Table 4. While one study found that Caucasian athletes were comparable to Middle African athletes [55], another study found that Middle African athletes were more likely to have the Black athlete variations than other Black populations [33]. In Riding et al. (2019) [33], abnormal TWI (8.5% vs. 2.4%), anterior TWI (13.4% vs. 3.3%), and inferior TWI (8.5% vs. 2%) were more prevalent in Middle African athletes than other Black athletes. In the noncontrolled study by Grace et al. (2015) [56], Middle African athletes had a high prevalence of TWI (17%), LVH (67%), ST elevation (47%), and percentage abnormal (71%).

##### Mixed-Race Athletes

Mixed-race athletes (defined as athletes with Black and Caucasian parents [28]) were only assessed in two papers. Malhotra et al. (2021) found that mixed-race athletes were more closely associated with Black athletes than Caucasian athletes [28], as detailed in Table 5. TWI was more common in Black athletes than mixed athletes but far less common in Caucasian athletes (12.6%, 8.6%, and 2.3% respectively). Mixed-race athletes were similar to Black athletes in inferior TWI (2% vs. 1.5%; *p* = 0.49) and lateral/apical TWI (0.6% vs. 1%; *p* = 0.33). The other paper had small numbers of athletes that were not the focus of assessment [39].

#### 3.4.2. Groups with Minimal Evidence to Suggest Variation from Caucasian Athletes

##### Arab Athletes

Arab athletes were assessed in seven papers, six of which assessed against other ethnicities [33,36,52,53,54] and one noncontrolled paper [57]. Findings from these papers are detailed in Table 6. In six of the papers, Arab athletes were found to be comparable to Caucasian athletes, having similar variation compared to nonathletes, with a similar number of athletes identified as abnormal (2.2–8.4% vs. 0–6.9%) across different criteria [33,36,52,53,54]. One paper that assessed only Arab athletes found high levels of abnormal ECGs (20.9%); however, this was based on the 2010 ESC recommendations [57].

##### Asian Athletes

Asian athletes were assessed in six papers, three studies with only Asian athletes [58,59,60] and three studies comparing Asian athletes to other ethnicities [22,38,44]. Findings from these papers are detailed in Table 7. Five studies found Asian athletes to be comparable to Caucasian athletes in terms of ECG features [22,38,44,58,59]. One recent study found 6.7% of Asian athletes (predominantly Chinese) had abnormal ECGs, primarily driven by anterior TWI beyond V2 in females, which the paper stated was four times more prevalent than a comparable Caucasian cohort [60]. Although this should be reproduced in a controlled study, the authors suggested that anterior TWI in Chinese females is perhaps a normal variation.

##### Pacific Islander Athletes

Pacific Islander athletes were assessed in four papers [38,44,61,62], summarized in Table 8. In one study, Melanesian athletes were reported to be twice as likely as other Pacific Islanders and Caucasian athletes to have TWI, comparable to Afro-Caribbean athletes [62]. In this study, 11 Pacific Islander groups were assessed, finding that 14% had LVH and 7.4% had right-ventricular hypertrophy (RVH), a similar prevalence to Caucasian athletes. Another paper assessed Pacific Islander athletes along with a variety of other non-Caucasian athletes (Asian, African, Aboriginal Australian, Torres Strait Islander, and Māori) and found that non-Caucasian athletes were more likely to have early repolarization [61]. The other two papers found Pacific Islander athletes to be comparable to Caucasian athletes [38,44].

##### Hispanic Athletes

Small numbers of Hispanic athletes were assessed in four papers and were found to be comparable to Caucasian athletes [38,39,40,44].

## 4. Discussion

### 4.1. Summary of Results

Black athletes are the most common athlete population that has been compared to Caucasian athletes. The Black athlete repolarization variant (J-point elevation and domed ST elevation followed by TWI in V1–V4) is a well-documented difference between populations. Potentially, there is some nuance within Black athlete populations, as indicated by the various Black athlete subgroups noted in this review. There is not yet sufficient evidence to recommend changes to the normal athlete criteria for other racial groups. However, further study of other ethnic populations is required, which may lead to further refinement of the athlete ECG interpretation criteria.

### 4.2. Black Athletes

The Black athlete repolarization variant is currently the only ethnic variation in the International Criteria for athlete ECG interpretation. The Black repolarization variant is reflected in most research into Black athletes found in this review [15,16,17,22,24,25,27,28,32,33,36,37]. However, there is a possibility that more differences should be included. Early repolarization, ST elevation, and LVH are noted in this review as more common in Black athletes [15,16,17,22,23,24,25,28,30,31,32,35,37], but these ECG features are now considered normal in all athletes by the International Criteria [1]. Other TWI outside V1–V4 in the lateral and inferior leads was also more common in Black athletes than other athletes, albeit only in small numbers [15,16,27,28,36]. While these ECG changes appear to be more common in Black athletes than Caucasian athletes, there is insufficient evidence to suggest that any further changes should be made to the interpretation of ECGs for Black athletes. This could, however, be a direction of further research into the Black athlete population.

### 4.3. Black Athlete Subgroups

Black athlete subgroups in African populations, in some cases, showed a greater variability than Black athletes who are not African. West African and Middle African athletes appeared in 13 papers in this review and, in most circumstances, had a greater prevalence of the Black athlete variations than other Black athletes. They were only directly compared in one paper [33], where West African and Middle African athletes were more likely to have LVH, RVH, and TWI than any other athlete population, including other Black athlete populations.

### 4.4. Mixed-Race Athletes

The mixed-race athlete population (with one Black and one Caucasian parent) may be more closely related to the Black athlete population [28]. Currently, mixed athletes are not viewed as a specific group in the International Criteria. Assessing the mixed-race athlete ECG is an important developing area.

### 4.5. Other Ethnicities

Athletes of other ethnicities included in this review had some variation from Caucasian athletes but with insufficient evidence to suggest a change in their interpretation. In terms of Arabic, Asian, Pacific Islander, and Hispanic athletes, in most circumstances, these athlete groups were found to be like Caucasian athletes in their ECG variation.

Arabic athletes have been reasonably well investigated, in comparison to both Black and Caucasian athletes. While there were some small trends toward a greater prevalence of TWI and abnormal ECGs compared to Caucasian athletes, these changes were not significant.

Studies of Asian athletes included in this review were generally found to be comparable to Caucasian athletes. However, one recent study [60], in a predominantly Chinese origin athlete population, found a greater prevalence of anterior TWI in females than in similar Caucasian cohorts.

Studies of Pacific Islander athletes found their ECG features generally to be equivalent to Caucasian athletes., although Melanesian athletes may have TWI prevalence at a similar rate to Black athletes [62].

Hispanic athletes, although not often studied specifically, were reported to have the same ECG patterns as Caucasian athletes.

Therefore, while there may be some athlete ECG variations in Arabic, Asian, Pacific Islander, and Hispanic athletes, these variations are not well established in the current research.

The broad definition in the current International Criteria of who qualifies as a “Black” athlete has relevance as it currently allows individual clinician judgment. In a non-Black athlete, TWI in leads V3 and V4 would be considered abnormal and prompt further investigation (i.e., echocardiography) to rule out cardiomyopathy. TWI is more of an objective finding than whether the athlete in question (e.g., mixed race) qualifies as “Black”, in which case TWI in leads V3 and V4 is considered a normal athlete finding.

### 4.6. Limitations

This review had several limitations. First, due to the variation in assessment of the ECGs, no meta-analysis was performed, limiting the applicability of results, particularly across papers. Secondly, due to the changes in the interpretation of athlete ECGs in the last decade, the criteria used to determine abnormality changed significantly across papers, limiting the applicability of percentage abnormal results. Thirdly, several papers in this review used similar athlete cohorts, as a number of studies were published from growing databases of athletes. Therefore, the number of unique athletes included in this population is unknown. Some of the studies only considered ethnicity in a univariate sense and, therefore, did not account for confounding factors, such as age, body composition, choice of sport, and level. For example, a multi-sport database that reported higher rates of abnormality in Black athletes may be biased if it did not account for the greater likelihood for Black athletes of playing a higher-risk sport such as basketball. Lastly, only papers written in English were included, which limited the number of papers included, particularly given the aim of the review to assess the impact of ethnicity. The gaps in research noted in this review may be covered by papers in languages other than English.

## 5. Conclusions

Ethnicity impacts the accurate interpretation of athlete ECGs. This impact is well understood in Caucasian and Black athletes but less clear in other ethnic groups. Within Black athletes, there may be some variation based on geographical origin. While there is some suggestion that other ethnicities may impact athlete ECG interpretation, there is insufficient evidence based on the current literature to suggest any modifications to the current criteria for ECG interpretation in athletes. Additional research comparing athletes of various under-represented ethnicities is required to further refine athlete ECG interpretation guidelines.

## Figures and Tables

**Table 1:** Proportions of ECG changes in Black athletes compared to Caucasian athletes.

Study	Total Athlete Participants	Black Athletes	Caucasian Athletes	*p*-Value
TWI				
Zaidi, 2013	675	22%	5%	0.0001
Sheikh, 2013	5505	22.8%	4.5%	0.001
Rawlins, 2010	440	14%	2%	0.001
Papadakis, 2011	2723	22.8%	3.7%	0.0001
Malhotra, 2020	11,168	10.6%	2.3%	0.0001
Magalaski, 2011	964	4.3%	1.5%	0.04
Magalaski, 2008	1959	2.6%	0.2%	0.05
Kervio, 2013	282	6.2%	0%	0.01
Basavarajaiah, 2008	750	12%	0%	0.0001
Malhotra, 2021	3000	13%	2.3%	0.0001
Anterior TWI beyond V2				
Wilson, 2012	1220	9.8%	0%	0.05
Sheikh, 2013	5505	14.3%	2.5%	0.001
Rawlins, 2010	440	14%	2%	0.001
Papadakis, 2011	2723	12.7%	1.9%	0.001
Malhotra, 2020	11,168	3.7%	0.6%	0.0001
Malhotra, 2021	3000	3.7%	0.6%	0.0001
Inferior TWI				
Sheikh, 2013	5505	6.1%	1.7%	0.001
Papadakis, 2011	2723	6%	1.7%	0.001
Malhotra, 2020	11,168	1.3%	0.5%	0.001
Riding, 2019	1698	2%	0%	S
Malhotra, 2021	3000	1.5%	0.5%	0.04
Lateral TWI				
Wilson, 2012	1220	6.1%	0%	0.05
Papadakis, 2011	2723	4%	0.3%	0.001
Malhotra, 2020	11,168	0.9%	0.2%	0.001
Sheikh, 2013	5505	2.4%	0.3%	0.001
Malhotra, 2021	3000	1%	0.2%	0.02
LVH				
Magalaski, 2011	964	9%	2.5%	0.001
Magalaski, 2008	1959	2%	0.2%	0.0001
Kervio, 2013	282	21%	10.3%	0.01
Basavarajaiah, 2008	750	68%	40%	0.001
Waase, 2018	519	39.6%	21.9%	0.001
ST elevation				
Zaidi, 2013	675	73.7%	61%	0.001
Sheikh, 2013	5505	49.5%	20.2%	0.0001
Rawlins, 2010	440	11%	1%	0.001
Papadakis, 2011	2723	63.2%	26.5%	0.001
Basavarajaiah, 2008	750	85%	62%	0.001
Muramoto, 2014	1114	32.9%	19.6%	0.005
Leo, 2011	641	20%	4%	0.01
Malhotra, 2021	3000	63.6%	48.4%	0.0001
ER				
Sheikh, 2013	5505	34.7%	21.1%	0.001
Miragoli, 2019	414	50%	17%	0.014
Waase, 2018	519	72.6%	58.3%	0.009
Noseworthy, 2011	879	OR 5.8 95% CI 3.54–9.61		0.001
Percentage abnormal				
Wilson, 2012	1220	18%	5.8%	0.001
Sheikh, 2014	5506	11.5%	5.3%	0.0001
Magalaski, 2011	964	18.1%	8.3%	0.001
Magalaski, 2008	1959	30%	15%	0.001
Fuller, 2016	874	22%	NR	NR
Riding, 2015	2491	10%	2.1%	0.0001
Riding, 2019	1698	4.7%	0%	0.001
Chandra, 2014	4081	57.7%	21.3%	0.001
Maillot, 2018	1030	OR 2.70 95% CI (1.63–4.48)		0.0001
RAE				
Wilson, 2012	1220	4.7%	0%	0.05
Malhotra, 2020	11,168	3.8%	0.3%	0.0001
LAE				
Malhotra, 2020	11,168	5.7%	0.9%	0.0001

Abbreviations: TWI, T-wave inversion; LVH, left-ventricular hypertrophy; ER, early repolarization; RAE, right-atrial enlargement; LAE, left-atrial enlargement; NR, not reported.

**Table 2:** Percentages of ECG changes in West African athletes.

Study	Total Athlete Participants	West African Athletes
TWI		
Schimeid, 2009	155	20%
Pambo, 2020	75	32%
Pambo, 2019	159	40%
Riding, 2019 *	1698	6.4%
Anterior TWI		
Schimeid, 2009	155	4.3%
Pambo, 2020	75	10.7%
Pambo, 2019	159	18.7%
Riding, 2019 *	1698	7.8%
Inferior TWI		
Schimeid, 2009	155	0.5%
Pambo, 2020	75	8%
Pambo, 2019	159	8.8%
Riding, 2019 *	1698	3.6%
LVH		
Ilodibia, 2021	77	40.3%
ST elevation		
Pambo, 2020	75	45.3%
Pambo, 2019	159	47%
Ilodibia, 2021	77	29.9%
ER		
Ilodibia, 2021	77	51.9%
Dome-shaped ST-Segment elevation with TWI		
Ilodibia, 2021	77	27.3%
Percentage abnormal		
Sokunbi, 2021	180	15%
Schmied, 2009	155	25.8%
Schmied, 2013	210	12.4%
Pambo, 2020	75	8%
Pambo, 2019	159	23.3%

Abbreviations: TWI, T-wave inversion; LVH, left-ventricular hypertrophy; ER, early repolarization. * Riding 2019 compared to Caucasian athletes and found a significant difference between West African and Caucasian athletes in all areas within the table.

**Table 3:** Proportions of ECG changes in African athletes compared to others.

Study	Total Athlete Participants	African Athletes	Caucasian Athletes	Arab Athletes	*p*-Value
TWI					
Riding, 2014	1175	13%	1%	2%	S
McClean, 2019 diagnostic accuracy	1304	7%		2.1%	0.001
Di Paolo, 2012	216	14%	3%		0.05
Anterior TWI					
McClean, 2019 prevalence	732	23.2%		10.3%	0.0001
McClean, 2019 diagnostic accuracy	1304	2.6%		1%	0.05
Lateral TWI					
McClean, 2019 prevalence	732	3.5%		1%	0.02
McClean, 2019 diagnostic accuracy	1304	3.3%		1.4%	0.05
LVH					
Di Paolo, 2012	216	89%	42%		0.001
ST Elevation					
Di Paolo, 2012	216	91%	56%		0.001
Percentage abnormal					
McClean, 2019 prevalence	732	15%		4.3%	0.001
McClean, 2019 diagnostic accuracy	1304	10.5%		6.1%	0.01
Riding, 2014	1175	20%	6.9%	8.4%	0.001

Abbreviations: TWI, T-wave inversion; LVH, left-ventricular hypertrophy; S, significant.

**Table 4:** Proportions of ECG changes in Middle African athletes compared to other Black athletes.

Study	Total Athlete Participants	Middle African Athletes	Other Black Athletes	*p*-Value
TWI				
Riding, 2019	1698	8.5%	2.4%	S
Grace, 2015	45	17%		
Anterior TWI				
Riding, 2019	1698	13.5%	3.3%	S
Inferior TWI				
Riding, 2019	1698	8.5%	2%	S
LVH				
Grace, 2015	45	67%		
ST elevation				
Grace, 2015	45	47%		
Percentage abnormal				
Grace, 2015	45	71%		

Abbreviations: TWI, T-wave inversion; LVH, left-ventricular hypertrophy; S, significant.

**Table 5:** Proportions of ECG changes in mixed-race athletes compared to others.

Study	Total Athlete Participants	Mixed-Race Athletes	Black Athletes	Caucasian Athletes	*p*-Value (Black vs. Mixed)	*p*-Value (Caucasian vs. Mixed)
TWI						
Malhotra, 2021	3000	8.6%	13%	2.3%	0.0019	0.0001
Inferior TWI						
Malhotra, 2021	3000	2%	1.5%	0.5%	0.49	0.0039
Lateral TWI						
Malhotra, 2021	3000	0.6%	1%	0.2%	0.33	0.29
LVH						
Malhotra, 2021	3000	30%	17.6%	25.6%	0.0001	0.03

Abbreviations: TWI, T-wave inversion; LVH, left-ventricular hypertrophy.

**Table 6:** Percentages of abnormal ECGs in Arabic athletes compared to Caucasian athletes.

Study	Total Athlete Participants	Arabic	Caucasian
Wilson, 2012	1220	7.9%	5.8%
Riding, 2019	1698	2.2%	0%
Riding, 2015	2491	3.6%	2.1%
Riding, 2014	1175	8.4%	6.9%
McClean, 2019 prevalence	732	6.1%	
McClean, 2019 diagnostic accuracy	1304	4.3%	
Allattar, 2014	230	20.9%	

**Table 7:** Proportions of ECG changes in Asian athletes compared to others.

Study	Total Athlete Participants	Asian Athletes	Caucasian Athletes	Black Athletes
Anterior TWI				
Yeo, 2022	75 females	9.3%		
Percentage abnormal				
Ma, 2006	351	4.5%		
Kervio, 2013	282	10.3%	6.0%	21%
Drezner, 2016	5258	6.2%	4.2%	5.6%
Abu Bakar, 2018	100	5.2%		
Yeo, 2022	150	6.7%		
Le, 2010	658	47%	48%	47%

Abbreviations: TWI, T-wave inversion.

**Table 8:** Proportions of ECG changes in Pacific Islander athletes compared to others.

Study	Total Athlete Participants	Pacific Islander Athletes	Caucasian Athletes	Black Athletes
TWI				
Chatard, 2019	2281	1.8%	1.5%	
LVH				
Chatard, 2019	2281	12.9%	18.1%	
Percentage abnormal				
Drezner, 2016	5258	3.8%	4.2%	5.6%

Abbreviations: TWI, T-wave inversion; LVH, left-ventricular hypertrophy.

## Data Availability

The data presented in this study are available in the article and Supplementary Materials.

## Supplementary Materials

The following supporting information can be downloaded at: https://www.mdpi.com/article/10.3390/jcdd9060183/s1. Supplementary Appendix SA: Search Terms, Supplementary Appendix SB: Quality assessment; Supplementary Table S1. Study details; Supplementary Table S2: PRISMA checklist.
