# Supplementary material for: The Impact of Ethnicity on Athlete ECG Interpretation: A Systematic Review
Source: J Cardiovasc Dev Dis. 2022 Jun 8;9(6):183. doi: 10.3390/jcdd9060183 (PMC9225578; doi:10.3390/jcdd9060183)
Supplement: Supplementary file 1 [file jcdd-09-00183-s001.zip › jcdd-1763764-supplementary.pdf]

Supplementary Material

Supplementary Appendix SA: Search terms

MEDLINE

#	Searches
1	exp Athletes/
2	athlete*.mp.
3	player*.mp.
4	sportsmen.mp.
5	sportsperson.mp.
6	sportswomen.mp.
7	1 or 3 or 4 or 6
8	Ethnic Groups/
9	African American/
10	African American*.mp.
11	Arabs/
12	Arab*.mp.
13	Asian Americans/
14	Asian American*.mp.
15	Indigenous peoples/
16	(Indigenous or Aborign* or first nation*).mp.
17	exp Continental Population Groups/
18	race.mp.
19	origin.mp.
20	Hispanic Americans/
21	Hispanic American*.mp.
22	exp African Continental Ancestry Group/
23	(African American* or Afro-Caribbean*).mp.
24	Mixed-race.mp.
25	Oceanic Ancestry Group/
26	Pacific Islander*.mp.
27	Oceanic Ancestry Group*.mp.
28	exp Asian Continental Ancestry Group/
29	Asian Continental Ancestry Group*.mp.

30	South Asian ethnic group*.mp.
31	Ethnic*.mp.
32	8 or 9 or 10 or 11 or 12 or 13 or 14 or 15 or 16 or 17 or 18 or 19 or 20 or 21 or 22 or 23 or 24 or 25 or 26 or 27 or 28 or 29 or 30 or 31
33	Electrocardiography/
34	Electrocardiogra*.mp.
35	ECG.tw.
36	EKG.tw.
37	33 or 34 or 35 or 36
38	7 and 32 and 37

EMBASE

#	**Searches**
1	elite athlete/ or athlete/ or professional athlete/
2	athlete*.mp.
3	player*.mp.
4	sportsmen*.mp.
5	sportsperson*.mp.
6	sportswomen.mp.
7	1 or 2 or 3 or 4 or 5 or 6
8	ethnic group/
9	ethnic*.mp.
10	population group/
11	African American/
12	African American*.mp.
13	asian continental ancestry group/
14	asian/
15	african/
16	asain*.mp.
17	african*.mp.
18	indigenous people/
19	aboriginal*.mp.
20	exp oceanic ancestry group/

21	exp african caribbean/
22	exp Black person/
23	8 or 9 or 10 or 11 or 12 or 13 or 14 or 15 or 16 or 17 or 18 or 19 or 20 or 21 or 22
24	electrocardiography/
25	electrocardiogra*.mp.
26	ECG.tw.
27	EKG.tw.
28	24 or 25 or 26 or 27
29	7 and 23 and 28

Scopus

(TITLE-ABS-KEY (athlete* OR player* OR sportsmen OR sportswomen OR sportsperson) AND TITLE-ABS-KEY (ethnic* OR "african american*" OR arab* OR "asian american*" OR indigenous OR aborign* OR "first nation*" OR race OR origin OR "hispanic american*" OR "african american*" OR "afro-caribbean*" OR "mixed-race" OR "pacific islander*" OR "oceanic ancestry group" OR "asian continental ancestry group" OR "south asian ethnic group") AND TITLE-ABS-KEY (electrocardiogra* OR ecg OR ekg))

SPORTSDiscus

(athlete* OR player* OR sportsmen OR sportswomen OR sportsperson) AND (ethnic* OR "african american*" OR arab* OR "asian american*" OR indigenous OR aborign* OR "first nation*" OR race OR origin OR "hispanic american*" OR "african american*" OR "afro-caribbean*" OR "Mixed-race" OR "Pacific Islander*" OR "oceanic ancestry group" OR "asian continental ancestry group" OR "south asian ethnic group") AND (electrocariogra* OR ecg OR ekg)

Web of Science

(athlete* OR player* OR sportsmen OR sportswomen OR sportsperson) AND (ethnic* OR "african american*" OR arab* OR "asian american*" OR indigenous OR aborign* OR "first nation*" OR race OR origin OR "hispanic american*" OR "african american*" OR "afro-caribbean*" OR "Mixed-race" OR "Pacific Islander*" OR "oceanic ancestry group" OR "asian continental ancestry group" OR "south asian ethnic group") AND (electrocariogra* OR ecg OR ekg)

Supplementary Appendix SB: Quality assessment

Study ID	1	2	3	4	5	6	7	8	9	10	11	12	13	14	Final assessment
Ma 2006	Yes	Yes	Yes	Yes	No	No	No	NA	Yes	No	Yes	NA	NA	Yes	Fair
Zaidi 2013	Yes	Yes	Yes	Yes	No	No	No	NA	Yes	No	Yes	NA	NA	Yes	Fair
Wilson 2012	Yes	Yes	Yes	Yes	No	No	No	NA	Yes	No	Yes	NA	NA	Yes	Fair
Waase 2018	Yes	Yes	Yes	Yes	No	No	No	NA	Yes	No	Yes	NA	NA	Yes	Fair
Uberoi 2013	Yes	Yes	Yes	Yes	No	No	No	NA	Yes	No	Yes	NA	NA	Yes	Fair
Sokunbi 2021	Yes	Yes	Yes	Yes	No	No	No	NA	Yes	No	Yes	NA	NA	Yes	Fair
Sheikh 2014	Yes	Yes	Yes	Yes	No	No	No	NA	Yes	No	Yes	NA	NA	Yes	Fair
Sheikh 2013	Yes	Yes	Yes	Yes	No	No	No	NA	Yes	No	Yes	NA	NA	Yes	Fair
Schmied 2009	Yes	Yes	Yes	Yes	No	No	No	NA	Yes	No	Yes	NA	NA	Yes	Fair
Schmied 2013	Yes	Yes	Yes	Yes	No	No	No	NA	Yes	No	Yes	NA	NA	Yes	Fair
Riding 2015	Yes	Yes	Yes	Yes	No	No	No	NA	Yes	No	Yes	NA	NA	Yes	Fair
Riding 2019	Yes	Yes	Yes	Yes	No	No	No	NA	Yes	No	Yes	NA	NA	Yes	Fair
Riding 2014	Yes	Yes	Yes	Yes	No	No	No	NA	Yes	No	Yes	NA	NA	Yes	Fair
Rawlins 2010	Yes	Yes	Yes	Yes	No	No	No	NA	Yes	No	Yes	NA	NA	Yes	Fair

Rambarat 2021	Yes	Yes	Yes	Yes	No	No	No	NA	Yes	No	Yes	NA	NA	Yes	Fair
Papadakis 2011	Yes	Yes	Yes	Yes	No	No	No	NA	Yes	No	Yes	NA	NA	Yes	Fair
Pambo 2020	Yes	Yes	Yes	Yes	No	No	No	NA	Yes	No	Yes	NA	NA	Yes	Fair
Pambo 2019	Yes	Yes	Yes	Yes	No	No	No	NA	Yes	No	Yes	NA	NA	Yes	Fair
Noseworthy 2011	Yes	Yes	Yes	Yes	No	No	No	NA	Yes	No	Yes	NA	NA	Yes	Fair
Muramoto 2014	Yes	Yes	Yes	Yes	No	No	No	NA	Yes	No	Yes	NA	NA	Yes	Fair
Muramoto 2013	Yes	Yes	Yes	Yes	No	No	No	NA	Yes	No	Yes	NA	NA	Yes	Fair
Miragoli 2019	Yes	Yes	Yes	Yes	No	No	No	NA	Yes	No	Yes	NA	NA	Yes	Fair
McClean 2019	Yes	Yes	Yes	Yes	No	No	No	NA	Yes	No	Yes	NA	NA	Yes	Fair
McClean 2019	Yes	Yes	Yes	Yes	No	No	No	NA	Yes	No	Yes	NA	NA	Yes	Fair
Malhotra 2021	Yes	Yes	Yes	Yes	No	No	No	NA	Yes	No	Yes	NA	NA	Yes	Fair
Malhotra 2020	Yes	Yes	Yes	Yes	No	No	No	NA	Yes	No	Yes	NA	NA	Yes	Fair

Maillot 2018	Yes	Yes	Yes	Yes	No	No	No	NA	Yes	No	Yes	NA	NA	Yes	Fair
Magalski 2011	Yes	Yes	Yes	Yes	No	No	No	NA	Yes	No	Yes	NA	NA	Yes	Fair
Magalski 2008	Yes	Yes	Yes	Yes	No	No	No	NA	Yes	No	Yes	NA	NA	Yes	Fair
Luijkx 2012	Yes	Yes	Yes	Yes	No	No	No	NA	Yes	No	Yes	NA	NA	Yes	Fair
Leo 2011	Yes	Yes	Yes	Yes	No	No	No	NA	Yes	No	Yes	NA	NA	Yes	Fair
Kervio 2013	Yes	Yes	Yes	Yes	No	No	No	NA	Yes	No	Yes	NA	NA	Yes	Fair
Junttila 2011	Yes	Yes	Yes	Yes	No	No	No	NA	Yes	No	Yes	NA	NA	Yes	Fair
Jacob 2015	Yes	Yes	Yes	Yes	No	No	No	NA	Yes	No	Yes	NA	NA	Yes	Fair
Ilodibia 2021	Yes	Yes	Yes	Yes	No	No	No	NA	Yes	No	Yes	NA	NA	Yes	Fair
Haddad 2013	Yes	Yes	Yes	Yes	No	No	No	NA	Yes	No	Yes	NA	NA	Yes	Fair
Grace 2015	Yes	Yes	Yes	Yes	No	No	No	NA	Yes	No	Yes	NA	NA	Yes	Fair
Fuller 2016	Yes	Yes	Yes	Yes	No	No	No	NA	Yes	No	Yes	NA	NA	Yes	Fair
DiPaolo 2012	Yes	Yes	Yes	Yes	No	No	No	NA	Yes	No	Yes	NA	NA	Yes	Fair
Dhutia 2016	Yes	Yes	Yes	Yes	No	No	No	NA	Yes	No	Yes	NA	NA	Yes	Fair
Crouse 2009	Yes	Yes	Yes	Yes	No	No	No	NA	Yes	No	Yes	NA	NA	Yes	Fair
Chatard 2019	Yes	Yes	Yes	Yes	No	No	No	NA	Yes	No	Yes	NA	NA	Yes	Fair

Brosnan 2015	Yes	Yes	Yes	Yes	No	No	No	NA	Yes	No	Yes	NA	NA	Yes	Fair
Basavarajaiah 2008	Yes	Yes	Yes	Yes	No	No	No	NA	Yes	No	Yes	NA	NA	Yes	Fair
Alattar 2014	Yes	Yes	Yes	Yes	No	No	No	NA	Yes	No	Yes	NA	NA	Yes	Fair
AbuBakar 2018	Yes	Yes	Yes	Yes	No	No	No	NA	Yes	No	Yes	NA	NA	Yes	Fair
Calore 2016	Yes	Yes	Yes	Yes	No	No	No	NA	Yes	No	Yes	NA	NA	Yes	Fair
Yeo 2022	Yes	Yes	Yes	Yes	No	No	No	NA	Yes	No	Yes	NA	NA	Yes	Fair

Key:

Question 1: Was the research question or objective in this paper clearly stated?

Question 2: Was the study population clearly specified and defined?

Question 3: Was the participation rate of eligible persons at least 50%?

Question 4: Were all the subjects selected or recruited from the same or similar populations (including the same time period)? Were inclusion and exclusion criteria for being in the study prespecified and applied uniformly to all participants?

Question 5: Was a sample size justification, power description, or variance and effect estimates provided?

Question 6: For the analyses in this paper, were the exposure(s) of interest measured prior to the outcome(s) being measured?

Question 7: Was the timeframe sufficient so that one could reasonably expect to see an association between exposure and outcome if it existed?

Question 8: For exposures that can vary in amount or level, did the study examine different levels of the exposure as related to the outcome (e.g., categories of exposure, or exposure measured as continuous variable)?

Question 9: Were the exposure measures (independent variables) clearly defined, valid, reliable, and implemented consistently across all study participants?

Question 10: Was the exposure(s) assessed more than once over time?

Question 11: Were the outcome measures (dependent variables) clearly defined, valid, reliable, and implemented consistently across all study participants?

Question 12: Were the outcome assessors blinded to the exposure status of participants?

Question 13: Was loss to follow-up after baseline 20% or less?

Question 14: Were key potential confounding variables measured and adjusted statistically for their impact on the relationship between exposure(s) and outcome(s)?

NA: Not applicable

Supplementary Tables

Supplementary Table S1: Study details

Study ID	Country	Ethnicities included	No. of athlete participants	Mean age (age range)	Male %
Ma 2006	Other: China	Han Chinese	351	13-34	48.4
Zaidi 2013	UK	Black and Caucasian	675	Black 21.8 SD 5.4<br>Caucasian 21.7 SD 4.6	Black 81<br>Caucasian 80.3
Wilson 2012	Other: Qatar	West Asian<br>Black<br>Caucasian	1220	22.6 SD 6	100
Waase 2018	USA	African American<br>Caucasian	519	24.8 SD 4.3	100
Uberoi 2013	USA	African American<br>Caucasian	85	Given by position	100
Sokunbi 2021	Other: Nigeria	Nigerian	180	14.46 SD 1.76	60
Sheikh 2014	UK	Black and Caucasian	5505	Black 22.2 SD 5.7<br>Caucasian 19.3 SD 5.4	Black 85.8<br>Caucasian 76.8
Sheikh 2013	Other: UK and France	Black and Caucasian	1232	16.4 SD 1.3	Black 74.5<br>Caucasian 81.4
Schmied 2009	Other: Alergia	African ethnic groups:<br>Bantu<br>Semitic-Hamitic<br>Mande<br>Mixed	155	16.4 SD 0.68	100
Schmied 2013	Other: Gabon	West African	210	18.6	100
Riding 2015	Other: Qatar	Arabic<br>Black<br>Caucasian	2491	Arabic 21.3 SD 5.4<br>Black 24.1 SD 5.0<br>Caucasian 24.3 SD 5.4	100
Riding 2019	Other: Qatar	Black (African-American/Caribbean,	1698	African-American/Caribbean 27.8 SD 2.9	100

		East African, Middle African, West African, South Americans)<br>Arabic (North African, West Asian)<br>Caucasian (South European)		East African 24.2 SD 4.9<br>Middle African 25.4 SD 4.5<br>West African 23.8 SD 4.5<br>South Americans 26.6 SD 4.5<br>North African 23.7 SD 5.3<br>West Asian 23.8 SD 4.9<br>South European 25.8 SD 5.3	
Riding 2014	Other: Qatar	Arabic, Black African, Caucasian	1175	Arabic 22.7 SD 5.9<br>Black African 24.6 SD 4.7<br>Caucasian 24.4 SD 5.4	100
Rawlins 2010	Other: UK and France	Black and Caucasian	440	Black 21 SD 4.6<br>Caucasian 20 SD 4.0	0
Rambarat 2021	USA	Black and Caucasian	329	18.68 SD 0.86	0
Papadakis 2011	Other: UK and France	Black and Caucasian	2723	Black 22.5 SD 5.0<br>Caucasian 17.4 SD 4.1	100
Pambo 2020	Ghana	Ghanaian (Black West African)	75	19.1 SD 3.7	0
Pambo 2019	Ghana	Ghanaian (Black West African)	159	19.28 SD 2.9	100
Noseworthy 2011	USA	Caucasian<br>Black<br>Asian<br>Latino	879	18.4 SD 0.8	62
Muramoto 2014	USA	African American<br>Caucasian	1114	19.2 SD 1.6	56.7
Muramoto 2013	USA	African American<br>Caucasian	1114	19.2 SD 1.6	56.7
Miragoli 2019	Other: Italy	Black<br>Caucasian	414	13.6 SD 1.8	72
McClean 2019	Other: Qatar	Arab<br>Black	1304	Arab 15.9 SD 2.0<br>Black 15.2 SD 1.9	100

McClean 2019	Other: Qatar	Arab<br>Black	732	11-18	100
Malhotra 2021	UK	Mixed race (Black and Caucasian parents)<br>Caucasian<br>Black	3000	16.4 SD 1.3	100
Malhotra 2020	UK	Caucasian<br>Black	11168	Caucasian 16.4 SD 1.2<br>Black 16.7 SD 1.5	95
Maillot 2018	Other: France	Caucasian<br>Afro-Caribbean<br>Maghreb	1030	20	80.3
Magalski 2011	USA	Caucasian<br>Black	964	18-21	48
Magalski 2008	USA	Black<br>Caucasian<br>Other races	1959	23 SD 0.9	100
Luijkx 2012	Other: Netherlands	Caucasian Sub-Sahara African	38	23	100
Leo 2011	USA	Caucasian<br>African Americans	641	19.5 SD 1.5	54
Le 2010	USA	Caucasian<br>African American<br>Hispanic<br>Pacific Islanders	658	20	54
Kervio 2013	Other: Japan and France	Japanese<br>West -African Descent<br>"African-Caribbean"<br>Caucasian	282	24.1 SD 4.2	100
Junttila 2011	USA	Caucasian<br>Hispanic<br>African-American<br>Other	503	17-24	51

Jacob 2015	USA	Caucasian<br>Black<br>Biracial<br>Hispanic<br>Other	1755	Men 18.9 SD 1.3<br>Women 18.4 SD 1.4	49
Ilodibia 2021	Other: Nigeria	Nigerian, West African	77	Male athletes 25.5 SD 3.8<br>Female athletes 23.6 SD 3.1	55.5
Haddad 2013	USA	Black and Caucasian	129	Caucasian 19.8 SD 1.6<br>Black 19.4 SD 1.4	100
Grace 2015	Other: South Africa	Zulu descent	45	Boxers (Endurance (E)) 24 SD 2<br>Bodybuilders (Resistance (C))22 SD 4<br>Control (C) 22 SD 4	100
Fuller 2016	USA	Black<br>NonBlack	874	<18 to 21+	55
Drezner 2016	USA	Caucasian<br>African-American<br>Asian<br>Hispanic<br>Pacific Islanders<br>Native American<br>Other	5258	20.1	55
DiPaolo 2012	Italy, Switzerland, Algeria	African<br>Caucasian (Italian)	216	African 15.9 SD 0.7<br>Caucasian 16.5 SD 1.1	100
Dhutia 2016	UK	Caucasian, Black	8939	19 SD 5	72
Crouse 2009	USA	African-American<br>Caucasian	77	18 SD 1	100
Chatard 2019	Other: New Caledonia	Pacific Islanders:<br>New Caledonia<br>French Polynesia<br>Fiji	2281	19.4 SD 6.5	68.7

		Wallis and Futuna<br>Tonga<br>Tuvalu<br>Vanuatu<br>Papua New Guinea<br>Samoa<br>Cook, Salmon and Palau islands			
Chandra 2014	UK	Black and Caucasian	4081	19.5 SD 5.2	80.5
Calore 2016	Other: Italy and UK	Black and Caucasian	80	21 IQR 1-27	Black 75<br>Caucasian 74
Brosnan 2015	Australia	Described as Non-Caucasian vs Caucasian<br>Non-Caucasian includes Asian, African, Aboriginal Australian, Torres Strait Islander, Pacific Islander, Maori and other	726	Non-Caucasian 20.3 SD 3.6<br>Caucasian 19.9 SD 3.9	100
Basavarajaiah 2008	UK	Black athlete<br>Afro-Caribbean<br>West African<br>East African<br>Caucasian athlete<br>Black control	750	Black athlete 20.5 SD 5.8<br>Caucasian athlete 20.2 SD 4.9	100
Alaπttar 2014	Other: United Arab Emirates	Arabic	230	20.68 SD 5.22	100
AbuBakar 2018	Other: Brunei	Malay, Chinese, Dusun, Malay-Filipino,	100	25.2 IQR 9.0	90

		Malay-Chinese, and Malay-Indian			
Yeo 2022	Singapore	Chinese, Malay, Indian, Sikh, Indian-Chinese Indonesian	150	26.1 SD 5.7	50

PRISMA 2020 Checklist

Supplementary Table S2: PRISMA checklist

Section and Topic	Item #	Checklist item	Location where item is reported
**TITLE**			
Title	1	Identify the report as a systematic review.	1
**ABSTRACT**			
Abstract	2	See the PRISMA 2020 for Abstracts checklist.	2
**INTRODUCTION**			
Rationale	3	Describe the rationale for the review in the context of existing knowledge.	4
Objectives	4	Provide an explicit statement of the objective(s) or question(s) the review addresses.	4
**METHODS**			
Eligibility criteria	5	Specify the inclusion and exclusion criteria for the review and how studies were grouped for the syntheses.	5
Information sources	6	Specify all databases, registers, websites, organisations, reference lists and other sources searched or consulted to identify studies. Specify the date when each source was last searched or consulted.	5
Search strategy	7	Present the full search strategies for all databases, registers and websites, including any filters and limits used.	26-28
Selection process	8	Specify the methods used to decide whether a study met the inclusion criteria of the review, including how many reviewers screened each record and each report retrieved, whether they worked independently, and if applicable, details of automation tools used in the process.	5
Data collection process	9	Specify the methods used to collect data from reports, including how many reviewers collected data from each report, whether they worked independently, any processes for obtaining or confirming data from study investigators, and if applicable, details of automation tools used in the process.	5
Data items	10a	List and define all outcomes for which data were sought. Specify whether all results that were compatible with each outcome domain in each study were sought (e.g. for all measures, time points, analyses), and if not, the methods used to decide which results to collect.	5
	10b	List and define all other variables for which data were sought (e.g. participant and intervention characteristics, funding sources). Describe any assumptions made about any missing or unclear information.	5
Study risk of bias assessment	11	Specify the methods used to assess risk of bias in the included studies, including details of the tool(s) used, how many reviewers assessed each study and whether they worked independently, and if applicable, details of automation tools used in the process.	5
Effect measures	12	Specify for each outcome the effect measure(s) (e.g. risk ratio, mean difference) used in the synthesis or presentation of	5

Section and Topic	Item #	Checklist item	Location where item is reported
		results.	
Synthesis methods	13a	Describe the processes used to decide which studies were eligible for each synthesis (e.g. tabulating the study intervention characteristics and comparing against the planned groups for each synthesis (item #5)).	5
	13b	Describe any methods required to prepare the data for presentation or synthesis, such as handling of missing summary statistics, or data conversions.	5
	13c	Describe any methods used to tabulate or visually display results of individual studies and syntheses.	5
	13d	Describe any methods used to synthesize results and provide a rationale for the choice(s). If meta-analysis was performed, describe the model(s), method(s) to identify the presence and extent of statistical heterogeneity, and software package(s) used.	5
	13e	Describe any methods used to explore possible causes of heterogeneity among study results (e.g. subgroup analysis, meta-regression).	5
	13f	Describe any sensitivity analyses conducted to assess robustness of the synthesized results.	5
Reporting bias assessment	14	Describe any methods used to assess risk of bias due to missing results in a synthesis (arising from reporting biases).	5
Certainty assessment	15	Describe any methods used to assess certainty (or confidence) in the body of evidence for an outcome.	5
**RESULTS**			
Study selection	16a	Describe the results of the search and selection process, from the number of records identified in the search to the number of studies included in the review, ideally using a flow diagram.	6
	16b	Cite studies that might appear to meet the inclusion criteria, but which were excluded, and explain why they were excluded.	N/A
Study characteristics	17	Cite each included study and present its characteristics.	6
Risk of bias in studies	18	Present assessments of risk of bias for each included study.	29-30
Results of individual studies	19	For all outcomes, present, for each study: (a) summary statistics for each group (where appropriate) and (b) an effect estimate and its precision (e.g. confidence/credible interval), ideally using structured tables or plots.	6-17
Results of syntheses	20a	For each synthesis, briefly summarise the characteristics and risk of bias among contributing studies.	6-17
	20b	Present results of all statistical syntheses conducted. If meta-analysis was done, present for each the summary estimate and its precision (e.g. confidence/credible interval) and measures of statistical heterogeneity. If comparing groups, describe the	6-17

Section and Topic	Item #	Checklist item	Location where item is reported
		direction of the effect.	
	20c	Present results of all investigations of possible causes of heterogeneity among study results.	6-17
	20d	Present results of all sensitivity analyses conducted to assess the robustness of the synthesized results.	6-17
Reporting biases	21	Present assessments of risk of bias due to missing results (arising from reporting biases) for each synthesis assessed.	6-17
Certainty of evidence	22	Present assessments of certainty (or confidence) in the body of evidence for each outcome assessed.	6-17
**DISCUSSION**			
Discussion	23a	Provide a general interpretation of the results in the context of other evidence.	18-20
	23b	Discuss any limitations of the evidence included in the review.	20
	23c	Discuss any limitations of the review processes used.	20
	23d	Discuss implications of the results for practice, policy, and future research.	20
**OTHER INFORMATION**			
Registration and protocol	24a	Provide registration information for the review, including register name and registration number, or state that the review was not registered.	5
	24b	Indicate where the review protocol can be accessed, or state that a protocol was not prepared.	5
	24c	Describe and explain any amendments to information provided at registration or in the protocol.	N/A
Support	25	Describe sources of financial or non-financial support for the review, and the role of the funders or sponsors in the review.	20
Competing interests	26	Declare any competing interests of review authors.	N/A
Availability of data, code and other materials	27	Report which of the following are publicly available and where they can be found: template data collection forms; data extracted from included studies; data used for all analyses; analytic code; any other materials used in the review.	Supplementary Material

From: Page MJ, McKenzie JE, Bossuyt PM, Boutron I, Hoffmann TC, Mulrow CD, et al. The PRISMA 2020 statement: an updated guideline for reporting systematic reviews. BMJ 2021;372:n71. doi: 10.1136/bmj.n71

For more information, visit: http://www.prisma-statement.org/
